# Early career investigator biocommentary: Katherine Bline

**DOI:** 10.1038/s41390-024-03097-4

**Published:** 2024-02-20

**Authors:** Katherine E. Bline

**Affiliations:** 1 Center for Vaccines and Immunity, Nationwide Children’s Hospital, Columbus, OH USA; 2 Division of Critical Care Medicine, Nationwide Children’s Hospital, Columbus, OH USA


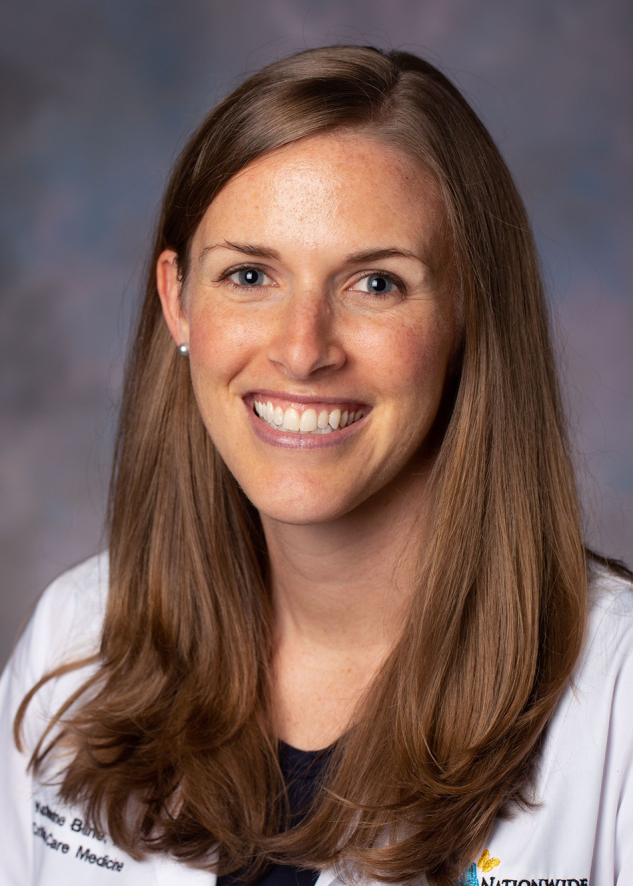
I am a pediatric intensivist at Nationwide Children’s Hospital in Columbus, OH, focused on the immune response to respiratory viruses in critically ill children. I grew up in Bloomington, Indiana before going to the University of Notre Dame for my undergraduate degree and attending medical school at the Ohio State University College of Medicine. Although I was fairly certain I wanted to pursue pediatrics early in medical school, it was not until I did a rotation in the pediatric intensive care unit as a fourth-year medical student that I knew that I had found my passion. I enjoyed the complex physiology of critically ill children, the procedures, and the opportunity to help patients and their families during one of the most difficult periods in their lives.

As a pediatric critical care fellow, I was struck by how often parents of patients asked me why their child was so sick from respiratory viruses that typically only cause symptoms of the common cold. These clinical experiences, as well as becoming a parent myself, really drive me to try to understand the immune dysregulation in children who become critically ill with respiratory viruses. I am particularly interested in the role of myeloid-derived suppressor cells in perpetuating immune suppression in children with critical illness and the impact of this cell population on clinical outcomes. My ultimate goal is to identify and develop more targeted treatments for this patient population. With the help of my incredible mentors, Dr. Mark Hall and Dr. Octavio Ramilo, I gained additional training in immunobiology and laboratory techniques during medical training and early faculty years to allow me to launch my career as a clinician-scientist.

I am extremely grateful to have a career that allows me to simultaneously pursue my two passions of working with critically ill patients and investigating questions that arise at the bedside to improve the clinical care we provide. For those who are considering the path of a clinician-scientist, my advice is to develop a supportive mentorship team, seek opportunities to collaborate, encourage the next generation of researchers, and enjoy the process.

